# Barriers and facilitators to pediatric tuberculosis management in India: a systematic review

**DOI:** 10.1186/s12879-025-10863-0

**Published:** 2025-04-10

**Authors:** Matthew Willis, Joelle Van de Wetering, Heynes Brown, Ewelina Julia Barnowska, Sophie CW Stuetzle, Mohammed Nadiruzzaman, Anil Fastenau

**Affiliations:** 1 School of Medicine, Dentistry & Biomedical Sciences, Queen’s University Belfast, Belfast, BT7 1NN UK; 2 Department of Health, Ethics & Society, Care and Public Health Research Institute CAPHRI, Faculty of Health, Medicine and Life Sciences, Maastricht University, Maastricht, The Netherlands; 3 Department of Global Health, Institute of Public Health and Nursing Research, University of Bremen, Bremen, Germany; 4 Marie Adelaide Leprosy Center, Karachi, Pakistan; 5 German Leprosy and Tuberculosis Relief Association (DAHW), Wuerzburg, Germany

**Keywords:** Tuberculosis, Pediatric, Systematic review, Tuberculosis management, Pediatric tuberculosis

## Abstract

**Background:**

Pediatric tuberculosis (TB) is a significant public health concern in India, contributing to 28% of the global pediatric TB burden. Despite its impact on child health, strategies to control TB have inadequately addressed pediatric cases, resulting in suboptimal management. Understanding the barriers and facilitators influencing the management of pediatric TB in India is crucial for improving healthcare practices and outcomes for affected children.

**Methods:**

This systematic review aims to analyze existing literature to identify barriers and facilitators to the management of pediatric TB in India. A search of three databases; Embase, PubMed and Web of Science, was conducted to identify relevant studies published from 01/01/2012 to 07/09/2024. Studies focusing on barriers and facilitators in pediatric TB management were included in this review.

**Results:**

The search strategy yielded 1132 original articles, with 24 articles meeting the selection criteria for inclusion. In this review, the prevalent barrier at the individual level encompasses parental knowledge gaps, financial constraints, and the preference for private healthcare services. At the community level, barriers included gender inequality, stigmatization and discrimination, interconnected living environment, and traditional practices. At the health system level, the most significant barriers included the insufficient knowledge and training among healthcare providers, diagnostic complexities, non-child-friendly tools and techniques, limited availability and accessibility of resources, inadequate monitoring, and practices in the private healthcare sector. The facilitators identified in this review included the collaboration between different sectors and the utilization of the Xpert MTB/RIF diagnostic test.

**Discussion:**

This systematic review provides a comprehensive understanding of the multifaceted challenges influencing the management of pediatric TB in India. By identifying the barriers and facilitators at different levels, the review offers valuable insights into the complexities of the diagnosis, treatment, and prevention of pediatric TB. A better understanding of these complexities is essential for improving pediatric TB management in India and worldwide.

**Supplementary Information:**

The online version contains supplementary material available at 10.1186/s12879-025-10863-0.

## Introduction

Tuberculosis (TB), caused by Mycobacterium tuberculosis, remains a significant global health concern. The World Health Organization (WHO) reported approximately 10.6 million TB cases worldwide in 2021 [1]. India, among the countries with the highest burden of TB, accounted for approximately 27% of the global TB cases [2]. Approximately 1.5 million children contract TB annually, representing 11% of the total global burden and 16% of the global TB-related deaths. India alone shoulders 28% of the global childhood TB burden [3, 4]. Despite this alarming statistic, the impact of TB on children was long disregarded, resulting in lower priority given to pediatric TB in the global TB response [5]. However, in recent years, growing evidence suggests that children are not only susceptible to TB, but also at a higher risk of developing severe or disseminated forms of the disease [6]. Only since 2011, international and national health actors have begun acknowledging children as a critical component of TB strategies. For instance, the WHO published the first global estimates for pediatric TB in 2012, followed by a roadmap for ending child TB by multiple international stakeholders in 2013. The 2015 WHO’s End TB Strategy marked the first time that children were explicitly included in TB strategies [4]. In India, the national TB program updated its guidelines in 2012 to incorporate pediatric TB management [7].

The National Tuberculosis Program (NTP), launched in the 1960s, aimed to establish India as a leader in the global effort of eliminating TB as a public health concern. However, since then, India has struggled to achieve the elimination of TB, and contrary to hopes at the time, the country continues to bear a high burden of TB and Multi Drug resistant (MDR) TB [2]. Still, the work of the program has taken steps in the right direction by strengthening public health services and launching the Revised National Tuberculosis Control Program (RNTCP). The RNTCP, now known as the National Tuberculosis Elimination Program (NTEP), has played an important role in India’s fight against TB by providing standardized guidelines and protocols for TB diagnosis, treatment, and prevention across the country [8, 9]. The guidelines encompass specific recommendations for the management of pediatric TB, recognizing the complexities involved in diagnosing and treating children [9]. While the guidelines have contributed to advancements in TB management, there remain challenges with the implementation of the guidelines at the ground level [9].

TB mortality and incidence have decreased across all age groups for both males and females over the period 1990–2019 [2]. Despite this success the neglect of the significance of pediatric TB for many years has caused the emergence of various challenges to its management. Pediatric TB often remains undiagnosed or is diagnosed late, leading to higher morbidity and mortality rates [10]. For instance, despite the advances in molecular diagnosis such as Xpert MTB-RIF test, some labs still rely on sputum smear microscopy, a commonly used diagnostic tool for TB in adults. This technique does not perform effectively in children, resulting in false-negative sputum tests and therefore contributing to the underestimated burden of pediatric TB [10, 11]. Furthermore, many of the concepts for the diagnosis of adult TB are redundant in a pediatric population where many cases are paucibacillary or extrapulmonary [12]. This further creates difficulties when considering the use of sputum positivity as a trigger for contact screening when considering the low sputum positivity of children with TB [11] and also the extrapulmonary nature of many pediatric TB cases which will not yield a positive sputum culture.

Many technological advancements in diagnosing TB in adults are not yet applicable to children, and research on pediatric drug formulations and the development of first- and second line medication lags behind [10]. Inadequate awareness among the general population regarding pediatric TB further hinders individuals from seeking care, as they lack knowledge and understanding of the disease’s signs and symptoms [13]. Moreover, the apprehension of encountering social stigma from family or community members may impede patients from seeking care or adhering to treatment [8]. The emergence of these challenges has led to barriers affecting timely diagnosis, appropriate treatment, and overall health outcomes for children with TB.

Pediatric tuberculosis poses a substantial burden in India, yet the research addressing the complexities of its management remains insufficient [14]. This research gap emphasizes the necessity for comprehensive insights into the factors that hinder or aid pediatric tuberculosis management in the country. The main objective of this systematic review is to identify and analyze the various barriers and facilitators associated with pediatric tuberculosis management in India. This study aims to provide a comprehensive understanding of the complex dynamics involved. Therefore, this research will examine the barriers and facilitators operating at the individual, community, and health system levels to elucidate how these interconnected factors might interact and influence the management of pediatric tuberculosis.

## Methods

A systematic literature review, following PRISMA guidelines, was carried out in order to collate the evidence available regarding facilitators and barriers to paediatric TB care in India with the final search taking place on the 07/09/2024 [15].

### Search strategy

The search strategy covered three online databases: PubMed, Embase and Web of Science, chosen for their different scopes and wide range of disciplines [16]. To develop the search query for PubMed, the eight chosen relevant preliminary search articles were utilized to extract MeSH Terms and free text terms. Subsequently, the query was then adapted to fit the required format for the Web of Science and Embase databases (Appendix 1) Rayyan software was used to manually remove duplicates and manage the results of the search strategy. Following this, a blinded screening of titles and abstracts was performed using Rayyan by MW, EB, and HB applying predefined selection criteria to determine the inclusion or exclusion of articles (Table 1) any paper receiving a “maybe” or “include” response by at least one author proceeded to full text screening. Full-text articles were then retrieved and assessed for eligibility using the criteria in Table 1. Table 2 in the result section contains more detailed information about the included articles. The PRISMA flow diagram visually represents the process of identifying, screening, and including relevant articles in this systematic review and can be found in the results section. The PRISMA checklist is attached in Appendix 2.

### Selection criteria

Included in this systematic review were articles focusing on pediatric tuberculosis management in Indian children aged 0 to 18 years, encompassing aspects related to diagnosis, treatment, or prevention. The review was limited to articles published between 2012 and the date of the search (07/09/2024), aiming to identify barriers and facilitators that emerged since the incorporation of pediatric components into TB strategies and guidelines by international and national actors, such as the 2012 Indian Revised National Tuberculosis Control Programme (RNTCP),2015 WHO’s End TB Strategy and the WHO guidelines for the management of tuberculosis in children and adolescents 2022 [7, 17, 18]. Non-peer reviewed articles were excluded, as were summaries, citations, editorials, commentaries, review articles, and case studies. All the selection criteria can be found in Table 1.

**Table 1:** Selection criteria

Inclusion Criteria
Articles should be written in English
The articles should be peer-reviewed journal articles
The articles should be published between 2012 to 0709/2024
The study population should be in India
The study should address tuberculosis in children (0–18 years) or those who work solely with tuberculosis in children (0–18 years)
The study should address either management, diagnosis, treatment (adherence) or prevention
The study provides information on the barriers or facilitators to paediatric Tb management
**Exclusion Criteria**
Summaries, citations, editorials and commentaries
Non peer-reviewed journal articles
Review papers
Case studies
Articles where children are not the primary focus, but a subset of the study population
Articles that don’t have tuberculosis as primary focus
Articles that focus on multiple countries rather than specifically on India

**Table 2:** Included studies

#	Author/ Publication Year	Title	Focus	Study Design	Method	Study setting	Study population	Barriers identified at		
								Individual level	Community level	Health system level
1	Belgaumkar et al., 2018	Barriers to screening and isoniazid preventive therapy for child contacts of tuberculosis patients	Prevention	Cross sectional study	Semi structured questionnaire, health record data	Sassoon General Hospital, Pune	Household child contacts (*n* = 178)	x	x	x
2	Bhat et al., 2013	Intensified tuberculosis case finding among malnourished children in nutritional rehabilitation centres of Karnataka, India: Missed opportunities	Prevention	Cross sectional study	Review of routinely collected program data and medical records	Nutritional Rehabilitation centers in 6 tehsils in Karnataka	Children with severe malnutrition (SAM) (*n* = 1927)	x	x	x
3	Chawla et al., 2023	Tuberculosis screening for pediatric household contacts in India: Time to adapt newer strategies under the National TB Elimination Programme!	Prevention	Cohort Study	Enrolled contacts of index TB cases were followed up at regular intervals for one year.	Bengaluru and Udupi districts of Karnataka, India	Household child contacts aged < 15 years (*n* = 686)	x	x	x
4	Chawla et al., 2021	Challenges perceived by health care providers for implementation of contact screening and isoniazid chemoprophylaxis in Karnataka, India	Prevention	Qualitative Study	In-depth interviews	Districts of Bengalaru, Udupi, Karnataka	Healthcare providers *n* = 64	x	x	x
5	Das, M. et al., 2021	Challenging drug-resistant TB treatment journey for children, adolescents and their care-givers: A qualitative study	Treatment	Qualitative study	In-depth interviews	Sassoon General Hospital, Pune	Adolescents (*n* = 6) Patient guardians (*n* = 5) Health workers (*n* = 8)	x	x	x
6	De et al., 2014	Source case detection for Children with TB Disease in Pune, India	Prevention	Cross sectional study	Questionnaire to legal parent or guardian	Sassoon General Hospital, Pune	Paediatric index cases (*n* = 50)	x	x	x
7	Dhaked et al., 2018	Treatment seeking pathways in pediatric tuberculosis patients attending DOTS centers in urban areas of Delhi*	Treatment	Cross sectional study	Semi-structured questionnaire to caregivers	Two chest clinics (DOTS & DMCs) located in Delhi	Pediatric TB patients (*n* = 141)	X	X	X
8	Dhaked et al., 2019	Socio-demographic profile and treatment outcomes in pediatric TB patients attending DOTS centers in urban areas of Delhi*	Treatment	Prospective study	Semi-structured questionnaire to caregivers	Two chest clinics (DOTS & DMCs) located in Delhi	Pediatric TB patients (*n* = 141)		X	
9	Dhakulkar et al., 2021	Treatment outcomes of children and adolescents receiving drug-resistant TB treatment in a routine TB programme, Mumbai, India	Treatment	Descriptive study	Routine program data	Shatabdi Hospital, Mumbai	Pediatric DR-TB patients (*n* = 268)	X	X	X
10	Giridharan, P et al., 2023	Time Elapsed from onset of symptoms to antituberculosis treatment in children with central nervous system tuberculosis in a tertiary hospital in South India: A mixed-methods pilot study	Treatment	Mixed-methods pilot study	Semi-structured pretested questionnaire In-depth interview	Tertiary hospital in South India	Children up to 12 years of age, diagnosed with CNS-TB, and initiated on antitubercular treatment (*N* = 38)	X		X
11	Jain et al., 2013	Pediatric Tuberculosis in Young Children in India: A Prospective Study	Diagnosis	Prospective study	History, physical examination	Byramjee Jeejeebhoy Government Medical College, Pune	Children < 5 with suspected TB (*n* = 223)	X		X
12	Kalra, 2017	Care seeking and treatment related delay among childhood tuberculosis patients in Delhi, India	Treatment	Cross sectional study	Interviews to parents/ guardians	8 RNTCP DTCs across Delhi	Child TB patients (*n* = 175)	X	X	X
13	Kalra et al., 2020	Upfront xpert mtb/rif for diagnosis of pediatric tb-does it work? experience from India	Diagnosis	Project	Roll-out of Xpert MTB/RIF testing through capacity building and provider engagement	10 cities across India	Presumptive pediatric TB cases (*n* = 42,238)	X	X	X
14	McDowell et al., 2018	“Before Xpert I only had my expertise”: A qualitative study on the utilization and effects of Xpert technology among pediatricians in 4 Indian cities	Diagnosis	Qualitative study	Semi-structured interviews	Chennai, Delhi, Hyderabad & Kolkata	Pediatric physicians (*n* = 55)			X
15	Paradkar et al., 2019	Challenges in conducting trials for pediatric tuberculous meningitis: lessons from the field	Treatment	Clinical trial	Monthly trial reports, case report forms, registers	Tertiary referral hospitals in Chennai and Pune	Children with probable or confirmed TB (*n* = 3371)	X	X	X
16	Pathak et al., 2016	Can Intensified Tuberculosis Case Finding Efforts at Nutrition Rehabilitation Centers Lead to Pediatric Case Detection in Bihar, India?	Diagnosis, Prevention	Cohort study	Medical records, RNTCP registers and treatment logs	7 Nutritional Rehabilitation Centers in Bihar	SAM children (*n* = 440)			X
17	Raizada et al., 2018a	Accelerating access to quality TB care for pediatric TB cases through better diagnostic strategy in four major cities of India	Diagnosis	Project	Roll-out of Xpert MTB/RIF testing through capacity building and provider engagement	Chennai, Delhi, Hyderabad & Kolkata	Presumptive pediatric TB cases (*n* = 42,238)	X	X	X
18	Raizada et al., 2018b	Catalysing progressive uptake of newer diagnostics by health care providers through outreach and education in four major cities of India	Diagnosis	Project	Mapping, approaching, engagement of healthcare providers	Chennai, Delhi, Hyderabad & Kolkata	Pediatric healthcare providers (*n* = 3670)			X
19	Raizada et al., 2021	Pathways to diagnosis of pediatric TB patients: A mixed methods study from India	Diagnosis	Mixed methods	Semi-structured questionnaire	Chennai, Delhi, Hyderabad & Kolkata	Parents/guardians of child TB patients (*n* = 100)	X	X	X
20	Sahana et al., 2018	Management practices of tuberculosis in children among pediatric practitioners in Mangalore, South India	Diagnosis, treatment	Cross sectional study	Questionnaire	Mangalore	Registered pediatricians (*n* = 50)			X
21	Shivaramakrishna et al., 2014	Isoniazid preventive treatment in children in two districts of South India: does practice follow policy?	Prevention	Cross sectional study	Questionnaire	Krishnagiri and Tiravulur districts of Tamil Nadu	Household child contacts (*n* = 271)	X		X
22	Singh et al., 2017	Isoniazid Preventive Therapy among Children Living with Tuberculosis Patients: Is It Working? A Mixed-Method Study from Bhopal, India	Prevention	Mixed methods	Secondary data analysis, survey, interviews	Tuberculosis Unit, Bhopal	Household child contacts (*n* = 51), parents/caregivers of child contact (*n* = 14), healthcare providers (*n* = 11)	X		X
23	Singh et al., 2021	Poor adherence to TB diagnosis guidelines among under-five	Diagnosis, prevention	Mixed methods	NRC records, focus groups, interviews	NRCs in Sagar and Sheopur	SAM children (*n* = 3230), healthcare providers (focus			X
24	Valvi et al., 2019	Delays and barriers to early treatment initiation for childhood tuberculosis in India	Diagnosis, treatment	Cross sectional study	Questionnaire, hospital records	Byramjee Jeejeebhoy Government Medical College and Sassoon General Hospital, Pune	Children on anti-TB treatment (*n* = 89)	X		X

### Data extraction and analysis

Included studies underwent a detailed examination to extract relevant information. The extracted data, entailing various study characteristics such as the year of publication, study design, study population, and study setting, were systematically organized in a matrix. Following the social-ecological model [19], a set of codes was developed to categorize the data into the different levels: individual, community and health system. Additionally, the data was further classified based on the categories of diagnosis, treatment, and prevention. Within these categories, sub-themes representing common barriers reported in multiple articles were identified.

### Risk of bias

In keeping with PRISMA guidelines each paper was screened for bias, this was carried out independently for all papers by MW, EB and HB, with disagreements then discussed and resolved. The results are summarised in Appendix 3. Due to the diverse nature of papers included, the authors utilised three separate risk of bias tools to assess the data: BMJ AXIS; CASP; or the MMAT [22, 23, 24]. The checklists were assigned independently by MW, EB and HB before consensus was reached with all authors and are summarised in Appendix 3. Nine papers were deemed suitable for assessment with the BMJ AXIS checklist; six with the MMAT; three with CASP Qualitative; four with CASP Cohort; and two with CASP diagnostic.

## Results

**Figure 1:**
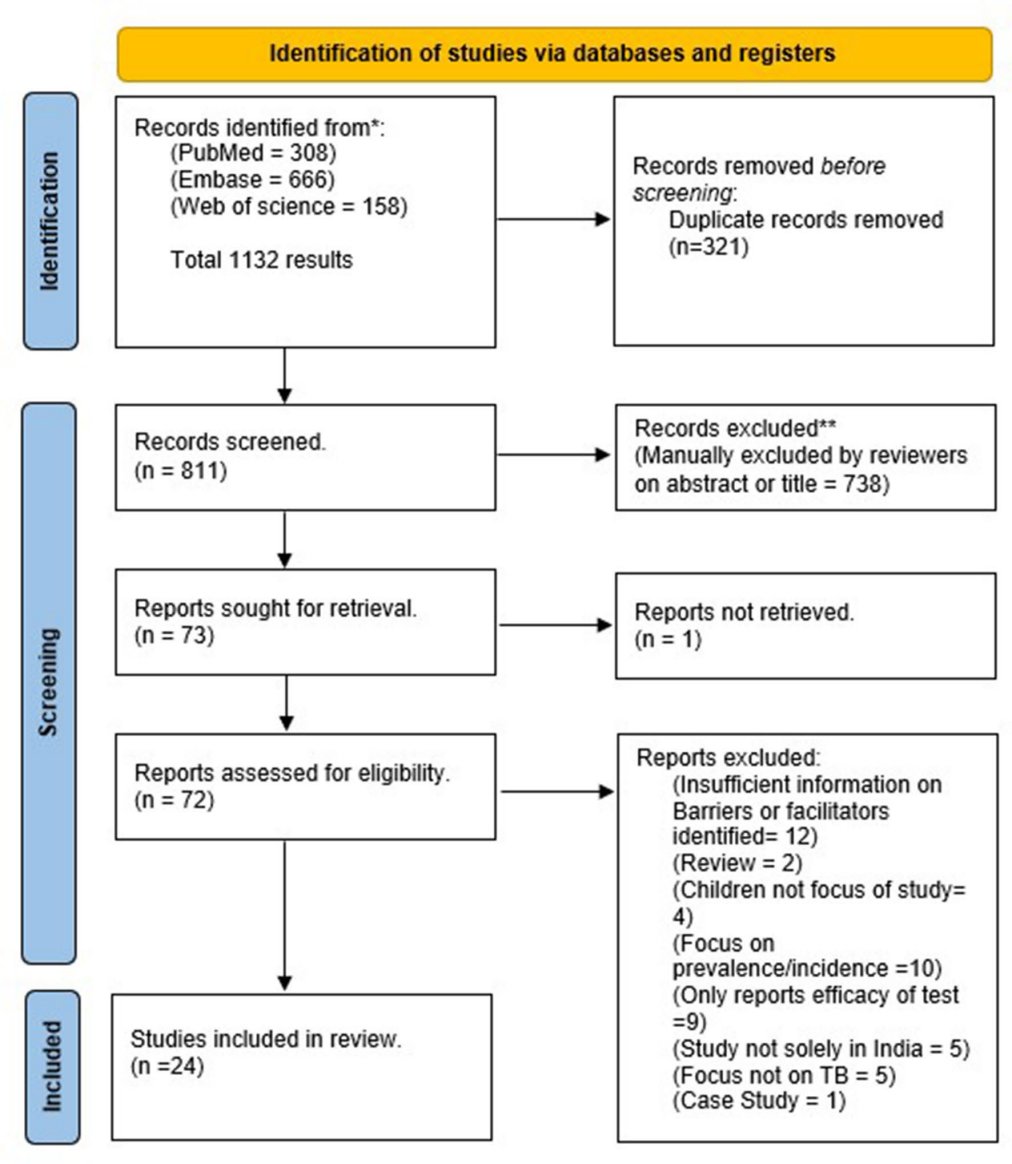
Fig. 1

The initial database search identified 1132 papers. After de-duplication (removing 321 papers), title and abstract screening (removing 738 papers), and full-text screening of 72 papers, 24 papers were found eligible for inclusion in this analysis. (Fig. 1).

The barriers and facilitators to the management of pediatric tuberculosis in India are divided into three major categories following the socio-ecological model [19]. These categories include the individual level, the community level, and the health system level. The facilitators to the management of pediatric TB were identified at the health system level. A summarized overview of the identified barriers and facilitators can be found in Table 3.

**Table 3:** Overview of the barriers and facilitators to pediatric tuberculosis management

SEM level	Barriers (-) / facilitators (+)	
Individual level	-	Lack of knowledge/awareness among parents
	-	Lack of financial resources
	-	Parents’ preference for private sector
	-	Lack of transportation
Community level	-	Gender inequality
	-	Stigmatization and discrimination
	-	Interconnected living environment
	-	Traditional practices
Health system level	-	Lack of knowledge and training among HCP
	-	Non-specific symptoms and challenges in sample collection
	-	Non-child-friendly tools, methods or procedures
	-	Limited availability and accessibility of resources
	-	Lack of monitoring
	-	Practices in the private sector
	+	Collaboration between various sectors
	+	The Xpert MTB/RIF diagnostic tes

### Methodological quality of included papers

The methodological quality of the papers included are summarised in Appendix 3. Overall, the results of the methodological assessment found that the methodological quality used in the papers identified was of good order for each class of intervention used. However, in some categories identified, the quality of methodological rigour was less thorough. This was particularly the case for one of the studies evaluated by the MMAT tool as one paper only obtained a definitive score of ‘yes’ in two of the seven categories available.

### Barriers at individual level

A barrier to managing pediatric tuberculosis at the individual level is the lack of knowledge/awareness among the patients and/or parents. Three articles highlight the lack of knowledge/awareness concerning the diagnosis of pediatric TB [25, 26, 27]. According to Kalra [23], only a small proportion of the parents understood that the symptoms were linked to TB. The lack of knowledge/awareness among the patient and/or parents was also apparent during the treatment of the disease [23, 27, 28, 29, 30]. In the study of Kalra [23], parents believed that the symptoms would naturally resolve without any treatment, indicating a general lack of knowledge/awareness about TB. Concerning the prevention of TB, four articles reported the lack of knowledge/awareness among the patients and parents [27, 31, 32, 33]. Parents were unaware about the necessity and advantages of preventive therapy for household contacts, leading to difficulties in making informed decisions about initiating treatment [30].

A second barrier identified at the individual level was the lack of financial resources. Five articles reported this issue as a barrier to the treatment of pediatric TB [23, 26, 28, 32, 33]. High treatment expenses at private healthcare facilities and the loss of income due to caring for the child during treatment were recognized as significant financial barriers [23, 32]. Regarding prevention, an additional four articles mentioned the lack of financial resources as a barrier on the individual level [29, 36, 37, 38]. Missing out on a day’s earnings to bring their child to a facility for a preventive check-up was one of the primary barriers to the prevention of pediatric TB on an individual level [25, 29, 34, 35].

Another barrier at the individual level to managing pediatric tuberculosis is the parents’ preference for the private sector in terms of diagnosis, treatment, and prevention [23, 25, 26, 29]. Several articles have revealed that parents tend to prefer opting for private healthcare facilities instead of government-run institutions, expressing greater trust in private providers [25, 26, 29].

Lastly, the lack of transportation could be a barrier at the individual level. Findings from two articles reported on transportation issues that hindered parents from bringing their child to a healthcare facility. Particularly in remote areas, commuting to and from facilities could be perceived as a challenge [29, 34].

### Barriers at community level

Gender inequality can be perceived as a community-level barrier to the management of pediatric TB, as indicated by various studies [24, 32, 37, 38]. These studies have reported a higher prevalence of TB among girls compared to boys. This disparity can suggest a gender disadvantage, where girls face several gender-related factors, including restricted access to healthcare, neglect in terms of vaccination and nutrition, and a lack of motivation to seek care for a girl by their caregivers, as reported in the studies [24, 32, 37, 38]. All these factors can contribute to the higher prevalence of TB among girls, highlighting gender inequality as a community-level barrier to the management of pediatric TB.

Another barrier at the community level is the stigmatization and discrimination concerning pediatric tuberculosis [24, 29, 32, 33, 35]. The stigma associated with tuberculosis prevents people from accessing healthcare, because they fear the social consequences such as exclusion and judgement that come with a TB diagnosis [29, 32]. Moreover, the stigma poses a hindrance for healthcare workers in performing their duties, as patients are reluctant to receive home visits out of fear that others might witness the presence of healthcare workers [29].

Having a densely interconnected living environment can be viewed as a barrier to the management of pediatric TB, particularly concerning its prevention [29, 35, 39]. The common extended family structure in India, combined with the close interactions with people beyond the family, such as neighbors and schoolchildren, creates challenges for healthcare workers in identifying and screening potential contacts of the TB index case [29, 39].

Lastly, a community-level barrier to the management of pediatric TB can be the preference for traditional practices in treating TB [23, 32, 36]. Three articles mention how patients and parents choose to consult traditional healers rather than seeking care at a government or even private healthcare facility. This preference is driven by personal or cultural beliefs, as well as by the proximity of the traditional healer’s location to their residence [23, 32, 36].

### Barriers and facilitators at health system level

One of the main barriers identified at the health system level is the lack of knowledge and training among healthcare providers (HCP). Six articles highlight the issue of HCP’s inadequate knowledge required for diagnosing pediatric TB [25, 42, 43, 44, 45, 46]. Specifically, their lack of knowledge regarding the correct use of the various diagnostic methods, unfamiliarity with the disease leading to the inability to recognize symptoms, and insufficient training on the RNTCP guidelines in terms of diagnosing methods hinder healthcare providers from efficiently diagnosing pediatric TB [40, 43, 44]. Moreover, five articles highlight the lack of knowledge among HCP about the treatment of pediatric TB [23, 25, 26, 36, 43]. Once again, HCP demonstrate limited understanding of the RNTCP guidelines concerning treatment [43]. Furthermore, ten articles report on the lack of knowledge among HCP regarding the prevention of pediatric TB [31, 32, 33, 34, 34, 36, 39, 41, 43, 44]. HCP’s lack of knowledge about the importance of home visits, contact screening, prescribing IPT to child contacts, and conducting follow-up visits, along with the insufficient training on the RNTCP guidelines regarding preventive practices, hinders the effective prevention of pediatric TB [39].

The health system-level barriers hindering the diagnosis of pediatric TB include the non-specific symptoms of the disease and the challenges associated with sample collection for the diagnosis [25, 26, 27, 29, 33, 36, 38, 40, 41]. Pediatric TB symptoms often resemble those of other childhood chest infections, leading to delays in clinical diagnosis when the symptoms are not correctly recognized [36]. Furthermore, the complexity and difficulty of sample collection pose challenges in obtaining a microbiologically confirmed TB diagnosis. Procedures like gastric lavage or induced sputum collection are reported to be challenging, resulting in low-quality specimens or even healthcare providers avoiding the procedure altogether, relying instead on the clinical presentation of the disease for diagnosis [38, 40]. The difficulty in collecting a sample is partly due to the non-child-friendly design of the collection methods [11].

Non-child-friendly tools, methods, or procedures pose a barrier at the health system level, impacting the diagnosis, treatment, and prevention of pediatric TB. Seven articles highlight that the diagnostic tools used to confirm tuberculosis are not specifically designed for the use in children [11, 25, 40, 41, 45, 46, 47], as children often struggle to provide adequate specimens for the tests [11]. In addition to the barriers encountered in diagnosis, non-child-friendly methods are also reported during the treatment phase [36, 41, 45]. Due to the lack of pediatric drug formulations, current medication is reported to be difficult to dose, and children encounter difficulties in consuming the medications due to issues with the size and taste of the drugs [36]. In addition, Singh et al. [31] mention the necessity for child-friendly dosage forms and a shorter treatment duration for the isoniazid preventive therapy to make it more suitable for children.

Another barrier identified at the health system level is the limited availability and accessibility of resources. Nine articles point out the lack of available or accessible diagnostic tests or tools at healthcare facilities to diagnose pediatric tuberculosis [24, 26, 27, 33, 36, 42, 43, 44, 44]. Often, due to the unavailability of these tools on-site, tests are outsourced to other facilities, causing delays in diagnosis [26, 41, 44]. The scarcity of resources also poses challenges to the treatment phase [32, 36, 45]. Drug supply shortages, lack of access to oral regimens for DR-TB treatment, and limited availability of pediatric formulations hinder the efficient treatment of pediatric TB [32, 36, 45]. Furthermore, the availability of resources presents obstacles for the prevention of pediatric TB [27, 29, 31, 34, 41]. One of the primary barriers to the non-initiation and non-completion of the IPT was the non-availability of the necessary medicines [31].

The inadequate monitoring of pediatric TB presents a barrier at the health system level, affecting its diagnosis, treatment, and prevention. Four articles highlight the inadequate monitoring during the diagnosis process [24, 25, 38, 44]. Poor record-keeping of crucial information, including the patient’s TB history, referrals to multiple facilities, drug resistances, contributes to the deficiency in monitoring, resulting in an information gap about the patient that is necessary for an accurate diagnosis [38, 44]. Three articles reported the lack of monitoring during treatment [23, 32, 45]. According to Das et al. [32] the inadequate monitoring can be attributed to the fact that patients receive minimal attention during follow-up visits unless they experience adverse or side effects of the treatment. Additionally, the monitoring tools used by the patients are difficult to use, leading to noncompletion and further complicating the monitoring process [32]. Moreover, the lack of monitoring also extends to the prevention phase, as reported in six articles [31, 32, 33, 35, 39, 44]. A major challenge for monitoring during prevention is the absence of a reporting mechanism for child contacts, which hinders access to crucial information, including details of the child contact, initiation and completion of IPT, referral sources, and other potentially exposed contacts, all of which are essential for HCP to effectively monitor and manage the prevention process [29, 31, 35, 39].

Lastly, the private sector can be perceived as a barrier at the health system level to managing pediatric TB [23, 25, 26, 40, 43]. There is a critical need for better engagement of the private sector to improve the management of pediatric TB, as several studies have reported suboptimal implementation and knowledge of the RNTCP guidelines among private providers [25, 26, 43]. The non-adherence to RNTCP guidelines by private providers leads to variations in the approach to disease definition, diagnostic methods, and treatment options, resulting in delayed or missed diagnoses and inadequate treatment [25, 40].

While the private sector can be perceived as a barrier as mentioned above, the collaboration between the public and private sectors emerges as a facilitator in managing pediatric TB [24, 25, 40, 43]. By establishing shorter linkages and promoting closer collaboration between the private and public sectors, patients can be promptly referred to facilities capable of recognizing symptoms or equipped with the necessary tools for diagnosis, avoiding delays and ensuring early diagnosis and initiation of treatment [25]. Additionally, Dhakulkar et al. [45] highlight the potential benefits of collaboration between the national TB program and other national initiatives, including maternal and child health and nutritional programs. This collaboration can aid in the early identification of pediatric TB cases and can provide support throughout the treatment process.

Improving the uptake of the diagnostic TB test Xpert MTB/RIF (Mycobacterium tuberculosis complex (MTBC) and resistance to rifampin (RIF)) can be perceived as a facilitator at the health system level in managing pediatric TB [24, 33, 36, 38, 40, 45]. The Xpert MTB/RIF test offers several advantages compared to other diagnostic tests, including higher sensitivity, rapid turnaround times, and the ability to detect rifampicin resistant cases [24, 40]. Establishing the Xpert MTB/RIF as the primary diagnostic test for all pediatric presumptive TB cases, as recommended by the WHO and RNTCP, can reduce diagnostic delays as the rapidity of the test allows for same-day diagnosis and treatment initiation [24, 40, 45].

## Discussion

This systematic review aimed to identify and analyze the existing literature on barriers and facilitators to the management of pediatric tuberculosis in India. Through an analysis of relevant studies, valuable insights are unveiled regarding the challenges and opportunities encountered in addressing pediatric TB. The findings shed light on critical aspects that influence the diagnosis, treatment, and prevention of the disease. Several common barriers were consistently reported across the studies. These included issues related to the healthcare system, such as inadequate training of healthcare providers, limited availability of child-friendly tools and methods, and the lack of monitoring. Additionally, community factors like stigma, traditional beliefs, and gender disparities emerged as critical challenges in the effective management of pediatric TB. Furthermore, individual barriers, such as the lack of knowledge among patients and parents about the disease, as well as financial constraints, hinder timely diagnosis and treatment-seeking behavior among patients and their families. Therefore, it can be said that the barriers to the management of pediatric TB are of multifaceted nature.

The findings across all SEM levels highlight this complex and multifaceted nature of challenges in managing pediatric TB. These multifaceted challenges are intricately interconnected, influencing one another and contributing to the overall complexity of the issue. For instance, the limited knowledge among parents or guardians may lead to misconceptions regarding pediatric TB, such as the belief that the symptoms will resolve without treatment [23]. In addition, the apprehension of social exclusion due to TB diagnosis, may prevent parents from disclosing the condition to neighbors or extended family members [29, 39]. In the close living proximity prevalent in India, this hesitation to share the TB diagnosis can lead to a heightened risk of infection among those in direct contact with a TB-infected individual. Unfortunately, many contacts remain unaware of the potential exposure and, as a result, miss out on necessary screening [29]. Furthermore, the study by Raizada et al. [33] found that many parents were unaware of the source of their child’s illness, resorting to blaming and suspecting other family members, school children, or neighbors. This lack of information and awareness about the disease contributes to the spread of TB within communities, as contacts are not receiving the appropriate preventive measures or timely medical attention. Consequently, this interconnectedness between the parents’ reluctance to disclose the diagnosis and the lack of awareness among potential contacts exacerbates the challenges in managing pediatric TB. Addressing these interconnected factors is crucial to improving the management of pediatric TB and reducing its impact on affected children and communities.

Another interconnected finding revolves around the preference of individuals to seek care in the private sector for pediatric TB management. This preference is rooted in the perception of higher trust and a desire for the best quality care that patients and parents associate with private providers [26, 29]. The interconnectedness becomes evident when considering the implications of this preference at both the individual and health system levels. At the individual level, parents’ trust in private providers leads them to initially seek care from the private sector, as indicated by the study conducted by Dhaked et al. [26]. However, the private sector’s management practices for pediatric TB may deviate from established guidelines like those of the NTEP (RNTCP) or WHO [25]. This lack of adherence to guidelines can result in varying definitions of the disease, different diagnostic methods, and non-standardized treatment options. Such divergence from recommended practices can contribute to misdiagnosis or inadequate treatment [25, 26]. Moreover, private providers often make late referrals of presumptive TB cases to TB clinics, causing delays in diagnosis and treatment initiation. As private providers are often the initial point of care, this delay further accentuates the interconnected challenge [25, 26, 43]. Therefore, the trust and preference for private providers at the individual level are intertwined with the shortcomings and non-standard practices at the private sector at the health system level. This connection highlights the complex interplay between patient choices and healthcare quality, underscoring the multifaceted nature of barriers in pediatric TB management.

Moreover, the role of healthcare providers in influencing pediatric TB management cannot be underestimated. Beyond their knowledge and training, their attitudes and communication skills can significantly influence the overall experience of patients and families seeking medical care. This lack of knowledge and training among healthcare providers can contribute to an overall lack of motivation. As illustrated in the study Singh et al. [44], healthcare providers lacked the motivation to carry out diagnostic tests to individuals with suspected TB. This lack of motivation might stem from an incomplete understanding of established guidelines or impracticality in performing certain diagnostic tests due to inadequate training [44]. Thus, the inadequate knowledge and training among healthcare providers can impact different aspects of pediatric TB management. It not only underscores the importance of comprehensive training for healthcare providers, but also highlights the interconnected nature of knowledge, attitudes and communication and their impact on the quality of care delivered.

It is evident that the pediatric TB situation in India is also exacerbated by the broader situation in the country with high rates of malnutrition and HIV infection leaving children susceptible to TB [11]. In addition, the definition of a contact used by the government to screen children has changed substantially, initially in 1997 the Indian academy of paediatrics defined this as a child who lives in a household with an adult receiving tb treatment within the last two years [46]. This was much broader than the current guidelines which have lessened the time of contact and limited to sputum positivity [7]. Another key factor in paediatric TB management not touched on in this review is the use of the BCG vaccine with the coverage at 1-year of age in India increasing from 74% in 2000 to 91% in 2022. Infant BCG vaccination is only 37% effective against all forms of tuberculosis in children younger than 5 years and 42% effective against pulmonary disease in children younger than 3 years [47]. Importantly however, it does not offer protection to adolescents or adults after close exposure [47]. Despite the clear evidence of success in preventing some of the most severe cases of childhood TB it is evident that its effect wanes in older children highlighting the need for the development of new screening and diagnostic tools for children in this older age bracket. In addition to the Xpert MTB-RIF test explored in the review there are also other tests not covered by this review being used in India such as the TruenatTM test which have shown to improve case detection and be operationally feasible under RNTCP [48]. This could hold potential for use in children in India with the test having been shown to aid in early and efficient diagnosis of TB in children in India [49].

Finally, although this review identified the individual factors contributing to the delay in pediatric TB identification and treatment, it is important to mention that the Indian government have put in place measures to try to relieve these burdens through the creation of the Accredited Social Health Activists (ASHA) in 2006 [50] and the Ayushman Bharath-Health and Wellness Centres (AB-HWCs) in 2018 [51]. The creation of these 2 resources intersect to try to alleviate some of the barriers mentioned in this review by reaching rural, and oftentimes marginalised, communities; as under the guidance of the Community Health Officer, ASHA workers are currently stationed within AB-HWCs in provinces across India [52]. Their work within these Health and Wellness Centres reduces the burden upon the individual patients, as they often alleviate individual motivation barriers for seeking treatment by generating awareness for TB screening within rural villages, through the use of home visits, among other means. The ASHA workers also aim to remove these individual barriers by completing Community Based Assessment Checklist forms and assisting with the collection and transportation of diagnostic samples to peripheral health institutes [52]. Moreover, the mandate for Directly Observed Treatment, Short Course (DOTS) as set out by the Revised National TB Control Programme (RNTCP) in 1997, is fulfilled by ASHA workers [53], however there are questions as to whether this is as closely followed in the private sector. As DOTS requires strict health policies to be effective, in areas where the quality of services have been shown to be deficient, such as within the private sector, questions may be raised as to whether these campaigns are as closely followed [54]. ASHA workers may also assist with maternal education surrounding the importance of following these DOTS [52], something which may be needed, as highlighted in this review.

### Limitations

While this systematic review has offered insights into the barriers and facilitators concerning pediatric TB management, it is important to address some limitations. This review did not differentiate or further specify the various types of tuberculosis, such as pulmonary TB, extrapulmonary TB, latent TB, DR-TB, or MDR-TB. This limitation could have implications for understanding how specific barriers may vary depending on the type of tuberculosis and its clinical manifestations. However, this choice was made due to the limited and inconsistent data across the included studies. Therefore, this review aimed to provide an overview of common overarching barriers and facilitators to managing pediatric TB. Further research with more comprehensive data could consider delving into the intricate nuances of barriers and facilitators associated with distinct types of pediatric tuberculosis. Furthermore, while barriers have been reported at all levels of the social-ecological model [19], the most frequently cited barriers were identified at the health system level. Addressing these limitations in future research could provide a more comprehensive understanding of the challenges and opportunities in managing pediatric TB effectively. This review was not registered with PROSPERO.

### Recommendations

Building upon the insights gained from this review on barriers and facilitators to pediatric TB management in India, several recommendations for research and practice have emerged. The facilitators identified in this review, namely the collaboration between different sectors and the utilization of the diagnostic test Xpert MTB/RIF, present opportunities to enhance pediatric TB management in India.

#### Strengthen inter-sectoral collaboration

The collaborative efforts between various sectors, including private and public healthcare providers, governmental departments, but also non-governmental organizations, and community stakeholders can emerge as a powerful facilitator in a holistic approach [25, 45]. Policymakers should support and incentivize inter-sectoral partnerships, encouraging knowledge exchange, resource sharing, and joint initiatives. Such collaborations can potentially address barriers at multiple levels, leading to more comprehensive pediatric TB management.

#### Amplify diagnostic utilization

The Xpert MTB/RIF diagnostic test has demonstrated its potential to enhance the pediatric TB diagnosis process by its rapid turnaround time and ability to detect rifampicin resistant cases [24, 40]. Healthcare systems should prioritize the integration and availability of the Xpert test within their diagnostic infrastructure [38]. Additionally, healthcare providers must be adequately trained in using the test to ensure its effective implementation [42]. This technology-driven approach can facilitate accurate diagnosis, enabling timely treatment initiation and reducing the transmission of the disease.

#### Capacity Building and training

Recognizing the pivotal role of healthcare providers in pediatric TB management, it is crucial to prioritize continuous and comprehensive training. This includes not only technical training on diagnostic tools like Xpert MTB/RIF but also focusing on effective communication and patient counseling [39]. By enhancing healthcare providers’ competencies, the quality of patient-provider interactions can be improved, contributing to better adherence, treatment outcomes, and overall patient satisfaction. Additionally, fostering ongoing medical education programs can keep healthcare providers updated on evolving practices and guidelines [43].

#### Community awareness and education

Interventions targeting community-level barriers such as stigma, traditional beliefs, and gender disparities are essential. Health education campaigns aimed at raising awareness about pediatric TB and reducing stigma should be developed and implemented [29, 35]. These efforts could potentially foster community support and encourage early diagnosis and treatment seeking behaviors. Additionally, educational programs should be designed to improve knowledge about the disease, its symptoms, treatment protocols and the importance of screening among the general population [35].

#### Strengthening health system resources

Given the identified barriers related to limited availability of resources, lack of child-friendly tools, and inadequate monitoring, investing in health system resources is crucial. Research should be dedicated to the development of child appropriate diagnostic tools and drug formulations [32]. Furthermore, monitoring and reporting systems should be strengthened to effectively document the patient’s medical history, referrals, contact screening, and treatment progress [29, 35, 44]. This ensures that healthcare providers can closely monitor individual cases, make informed decisions, and adjust treatment plans as necessary. By establishing robust monitoring and reporting mechanisms, HCP can better identify potential challenges such as DR-TB, leading to more effective interventions for pediatric TB management.

#### Future research directions

This review advocates for upcoming research to focus on understanding the implications of TB comorbidities on pediatric TB management, alongside investigating the impact of specific tuberculosis variants, including DR-TB and MDR-TB. Furthermore, future research should emphasize delving deeper into the social aspects of tuberculosis and their implications for both individuals and communities. For instance, the implications of gender inequality on tuberculosis management should be investigated. A deeper exploration into the interplay between factors like societal influences, cultural norms, and gender disparities within the context of tuberculosis can provide insights into designing more effective and inclusive interventions. By exploring these areas, researchers could contribute to the development of more holistic and impactful strategies for pediatric tuberculosis management, and ultimately tuberculosis eradication.

## Conclusion

In conclusion, pediatric tuberculosis in India is a significant public health concern. The findings of this systematic review highlight the gravity of this issue, emphasizing the need for action. The high burden of pediatric TB in India, accounting for a substantial portion of the global pediatric TB cases, underscores the urgency of addressing the multifaceted challenges in the management of pediatric TB. The barriers highlighted in this review, identified at individual, community, and health system levels, demonstrate the complex nature of the issue and the need for integrated solutions. Promising facilitators, such as collaboration among sectors and innovative diagnostic approaches, provide hope for improved management. Hence, addressing pediatric TB requires a holistic and multifaceted strategy, involving healthcare providers, policymakers, communities, and the broader healthcare system. This systematic review provides valuable insights to guide future initiatives aimed at enhancing pediatric TB management in India and beyond.

## Electronic supplementary material

Below is the link to the electronic supplementary material.

Supplementary Material 1

## Data Availability

All important data and results are included in this manuscript or supplementary files, all research the paper is based on is publicly available.

## Abbreviations

TB: Tuberculosis
AB-HWCs: Ayushman Bharath-Health and Wellness Centres
ASHA: Accredited Social Health Activists
DOTS: Directly Observed Treatment, Short Course
WHO: World Health Organization
NTP: National Tuberculosis Programme
MDR TB: Multi Drug resistant Tuberculosis
RNTCP: Revised National Tuberculosis Control Program
PRISMA: Preferred Reporting Items for Systematic Reviews and Meta-Analysis
PROSPERO: International Prospective Register of Systematic Reviews
CASP: Critical Appraisal Skills Programme
MMAT: Mixed methods appraisal tool
BMJ AXIS: British Medical Journal Appraisal tool for Cross-Sectional Studies
