# Supplementary material for: Barriers and facilitators to pediatric tuberculosis management in India: a systematic review
Source: BMC Infect Dis. 2025 Apr 10;25:495. doi: 10.1186/s12879-025-10863-0 (PMC11983860; doi:10.1186/s12879-025-10863-0)
Supplement: Supplementary file 1 — Supplementary Material 1 [file 12879_2025_10863_MOESM1_ESM.docx]

Appendix 3 Search strategy

| **Database 07/09/2024** | **Search query** |
| --- | --- |
| **PubMed = 308 Results** | ("tuberculosis"[MeSH Terms] OR  "Antitubercular Agents"[MeSH Terms] OR  "Tuberculin Test"[MeSH Terms] OR  "tuberculosis"[Title/Abstract] OR "TB"[Title/Abstract] OR "Antituberculosis"[Title/Abstract] OR "antitubercular"[Title/Abstract]) AND  ("child"[MeSH Terms] OR  "adolescent"[MeSH Terms] OR  "pediatrics"[MeSH Terms] OR  "infant"[MeSH Terms] OR  "childhood"[Title/Abstract] OR "child"[Title/Abstract] OR  "pediatric*"[Title/Abstract] OR  "paediatric*"[Title/Abstract] OR  "adolescen*"[Title/Abstract] OR  "youth"[Title/Abstract]) AND  ("India"[MeSH Terms] OR  "India"[Title/Abstract] OR  "Indian"[Title/Abstract]) AND (“Barrier*”[Title/Abstract] OR “Facilitat*”[Title/Abstract] OR “enabl*"[Title/Abstract] OR “Challeng*”[Title/Abstract] OR “Opportunit*”[Title/Abstract])  (2012:2024[pdat]) |
| **Web of Science = 158 Results** | TS=(("tuberculosis" OR "Antitubercular  Agents" OR "Tuberculin Test" OR "tuberculosis" OR "TB" OR "Antituberculosis" OR "antitubercular") AND ("infant" OR "child*" OR "pediatric*" OR  "paediatric*" OR "adolescen*" OR "youth") AND ( "India" OR “Indian”) AND (“Barrier*” OR “Facilitat*” OR “Enabl*” OR “Challeng*” OR “Opportunit*” OR “Success*” OR “Unsuccess*” OR “Delay*”)) AND  (PY==("2012" OR "2013" OR "2014" OR "2015" OR "2016" OR "2017" OR "2018"  OR "2019" OR "2020" OR "2021" OR  "2023" OR "2022" OR “2024”)) |
| **Embase = 666 Results** | (exp 'tuberculosis'/ OR exp 'Antitubercular Agents'/ OR exp 'Tuberculin test'/ OR tuberculosis.ti,ab,kf. or TB.ti,ab,kf. OR Antituberculosis.ti,ab,kf. OR antitubercular.ti,ab,kf.) AND (exp 'juvenile'/ OR exp 'pediatrics'/ OR childhood.ti,ab,kf. OR child.ti,ab,kf. OR pediatric*.ti,ab,kf. or paediatric*.ti,ab,kf. OR adolescen*.ti,ab,kf. or youth.ti,ab,kf.) AND (exp 'India'/ OR India*.ti,ab,kf.) AND (Barrier*.ti,ab,kf. OR Challeng*.ti,ab,kf. OR Enabl*.ti,ab,kf. OR Opportunit*.ti,ab,kf. OR Success*.ti,ab,kf. OR Unsuccess*.ti,ab,kf. OR Delay*.ti,ab,kf.)  limit 1 to yr="2012 -Current" |
